# Review of infectious diseases in refugees and asylum seekers—current status and going forward

**DOI:** 10.1186/s40985-017-0065-4

**Published:** 2017-09-08

**Authors:** Andreas Halgreen Eiset, Christian Wejse

**Affiliations:** Department of Public Health, Aarhus University, Aarhus, Denmark

**Keywords:** Infectious diseases, Refugees, Asylum seekers, Migrants

## Abstract

An unprecedented rise in the number of asylum seekers and refugees was seen in Europe in 2015, and it seems that numbers are not going to be reduced considerably in 2016. Several studies have tried to estimate risk of infectious diseases associated with migration but only very rarely these studies make a distinction on reason for migration. In these studies, workers, students, and refugees who have moved to a foreign country are all taken to have the same disease epidemiology. A common disease epidemiology across very different migrant groups is unlikely, so in this review of infectious diseases in asylum seekers and refugees, we describe infectious disease prevalence in various types of migrants. We identified 51 studies eligible for inclusion. The highest infectious disease prevalence in refugee and asylum seeker populations have been reported for latent tuberculosis (9–45%), active tuberculosis (up to 11%), and hepatitis B (up to 12%). The same population had low prevalence of malaria (7%) and hepatitis C (up to 5%). There have been recent case reports from European countries of cutaneous diphtheria, louse-born relapsing fever, and shigella in the asylum-seeking and refugee population. The increased risk that refugees and asylum seekers have for infection with specific diseases can largely be attributed to poor living conditions during and after migration. Even though we see high transmission in the refugee populations, there is very little risk of spread to the autochthonous population. These findings support the efforts towards creating a common European standard for the health reception and reporting of asylum seekers and refugees.

**Electronic supplementary material:**

The online version of this article (10.1186/s40985-017-0065-4) contains supplementary material, which is available to authorized users.

## Background

In 2015, asylum applications in the EU+ region amounted to approximately 1.35 million—a record since data collection began in 2008 and more than twice the number of applications in 2014 [1]*.* The available evidence on health problems among asylum seekers and refugees is limited in general with the best documentation on infectious diseases and mental and maternity health and almost non-existing for chronic diseases and childhood illnesses [2, 3].

In the EU, a number of communicable diseases have been reported to spread in the refugee population including acute respiratory tract infections, louse-borne relapsing fever, cutaneous diphtheria, scabies, measles, meningococcal meningitis, shigellosis, typhoid fever, hepatitis A, tuberculosis, and malaria [4]. Across studies, tuberculosis—particularly latent—and hepatitis B are the most commonly reported diseases [5–7]. A recent study including only Syrian refugees found leishmaniasis, tuberculosis, hepatitis, and vitamin D insufficiency to be the most prevalent health concerns [8].

The disease epidemiology of the country of origin is sometimes used to allocate the individual asylum seeker to a specific screening programme in the receiving country [9]. While this could be a quick approach and possibly reliable for quota refugees, most asylum seekers arrive to their destination after a period in transit and have been subject to poor living conditions and changing disease epidemiology. Further, the asylum seekers can often be considered a subgroup in their home country and as such the estimate for the general population is not applicable. One example of this is the debated *healthy migrant effect* that hypothesises that those who migrate are in a favourable health and/or socio-economic condition compared to those who stay in the country of origin [10–12].

When a study focuses on a sub-population of migrants (e.g. asylum seekers), country of origin, the reason for migration, the migration process itself, and the resettlement conditions are just some of the important factors that may influence the health of migrants. Very few studies take this into account in the analysis or reporting [3]. In the following, we present a literature review of the infectious diseases of special interest in the current asylum seeker and refugee populations including studies on health system utilisation and screening strategies. We pay special attention to the reporting on the definition of migrants in each study: Whether or not the studies account for the type of migrant and country of origin in the reported analysis either by design or as a variable.

## Methods

We included original studies and reviews on infectious diseases in asylum seeker and refugee populations published between January 1, 2010, and July 3, 2016. Publications with a main objective specifically related to other migrant subgroups than asylum seekers or refugees were excluded, as were studies concerned with health literacy and education. Studies that did not specify sub-population of migrants were included as well. After consulting a librarian we applied the following search strategy in PubMed: *“[disease]”[MeSH Terms] AND “epidemiologic studies”[MeSH Terms] AND “refugees”[MeSH Terms] AND (“2010/01/01”[PDAT]*: *“2016/07/03”[PDAT]) AND “adult”[MeSH Terms]* where “[disease]” was substituted with each of the diseases commented on below. Furthermore, we searched references and conference abstracts for additional publications and unpublished material. Also included were a number of relevant reports from the European Centre for Disease Prevention and Control (ECDC) and WHO. For childhood diseases, we used the PubMed search strategy: *“[disease]”[MeSH Terms] AND “epidemiologic studies”[MeSH Terms] AND “refugees”[MeSH Terms] AND (“2010/01/01”[PDAT]*: *“2016/07/03”[PDAT])* where “[disease]” was substituted with “rubella”, “mumps”, “measles”, and “vaccine”, respectively. Studies that reported on several diseases were only included once; data on all diseases were extracted. The title and abstract were screened and included articles were retrieved and read in full. Articles excluded after screening or full read-through were categorised according to predefined criteria and data were extracted according to predetermined variables (see “Availability of data and materials”).

When reporting on the included studies, we subdivided the migrant population into “foreign-born”, “refugee”, “family-reunificated”, “asylum seeker” and “border-crosser” as appropriate. Where no details were given on the sub-population of migrants, we used the super-population term “migrants”.

Data management and a plot summarising the presented data were done using R [13]. The R-code along with the data set and codebook are freely available (see “Availability of data and materials”).

The review conforms to the PRISMA statement checklist [14] (see Additional file 1)*.*

## Results

**Fig. 1:**
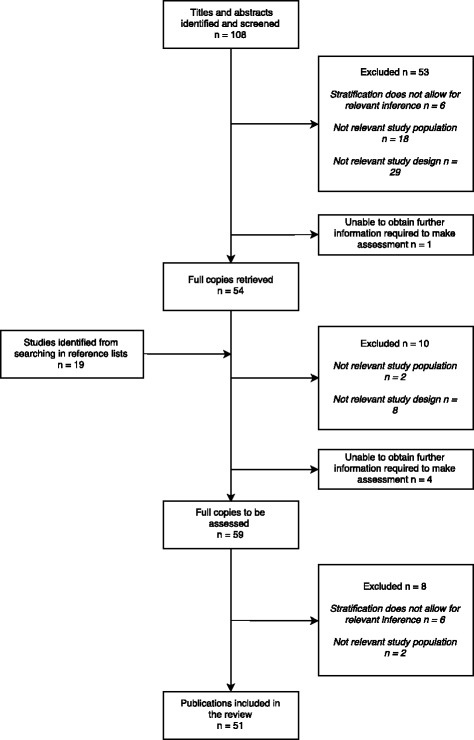
Flow chart of study selection

A total of 127 unique articles were identified and 51 of these included. A flow diagram of the combined searches including the number and reasons for exclusion is presented in Fig. 1. Of the included publications, the most commonly studied diseases were tuberculosis (29), hepatitis B (12), and HIV (8). Due to great heterogeneity, it was not possible to give a single measure for disease occurrence.

Eleven publications (23%) did not take into account the subgroup of migrant in the analysis and 12 studies (25%) did not stratify on ethnicity; three studies did neither [15–17]. Table 1 gives an overview of the included studies’ consideration of sub-population of migrant and country or region of origin in reporting their result.

**Table 1:** Migrant sub-population and ethnicity of study population accounted for—by design or inclusion of relevant variables—in the included studies’ analysis

	Migrant sub-population						Ethnicity		
Studies	No definition	Country of birth	Refugee	Family reunification	Asylum seeker	Border-crossing	No stratification	Country	World region
Pareek, 2016 [29]	X							X	
Odone, 2014 [18]		X						X	
Wallace, 2015 [68]		X						X	
Ikram, 2015 [32]		X							X
Greenaway, 2015 [55]		X	X	X	X				X
Rossi, 2012 [51]		X	X		X				X
Aldridge, 2014 [34]		X	X	X	X		X		
Norredam, 2012 [69]			X	X					X
Alawieh, 2014 [62]			X					X	
Padovese, 2014 [21]			X		X		X		
Trovato, 2016 [70]	X								X
Mor, 2013 [16]	X						X		
Bennett, 2014 [22]			X						X
Paulino, 2016 [37]		X							X
Ködmön, 2016 [33]		X							X
Raisanen, 2015 [31]	X							X	
Cookson, 2015 [42]			X					X	
Varughese, 2014 [17]	X						X		
Bradby, 2015 [3]			X		X		X		
Bozorgmehr, 2016 [2]			X		X		X		
Karki, 2014 [15]	X						X		
Barnett, 2012 [5]						X		X	
McCarthy, 2013 [7]						X		X	
Mockenhaupt, 2016 [8]			X					X	
Clark, 2007 [45]			X		X		X		
Eonomopoulou, 2016 [20]	X							X	
Evlampidou, 2016 [54]		X						X	
Angeletti, 2016 [43]					X		X		
Hargreaves, 2014 [36]		X						X	
Heidrich, 2014 [48]	X								X
Fasano, 2012 [53]	X								X
Contini, 2012 [52]	X							X	
Tafuri, 2010 [46]					X				X
Pérez-Molina, 2011 [49]	X								X
Museru, 2010 [50]			X				X		
Araj, 2016 [19]		X					X		
Nuzzo, 2015 [71]			X					X	
Kowatsch-Beyer, 2013 [38]			X						X
Simpson, 2012 [39]		X	X					X	
Sarivalasis, 2012 [28]					X				X
Trauer, 2011 [41]			X						X
CDC, 2010 [27]			X					X	
Schneeberger Geisler, 2010 [26]					X		X		
Harstad, 2010 [72]					X				X
Mendelsohn, 2014 [47]			X					X	
Stauffer, 2012 [44]			X						X
Rungan, 2013 [58]			X						X
Total	11	12	21	3	7	2	12	17	18

**Fig. 2:**
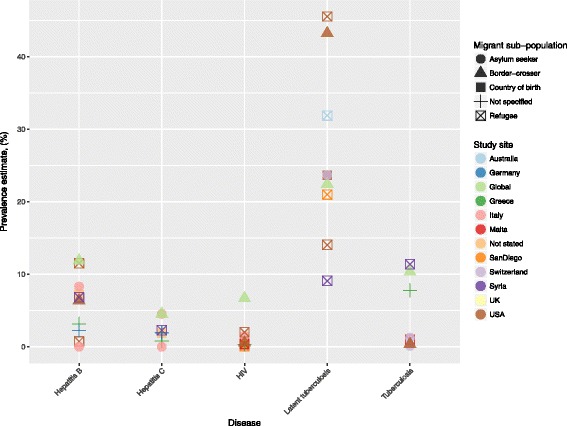
Prevalence estimates as reported in the included studies of a number of the infectious diseases of importance in the refugee and asylum seeker population in Europe in the 2010s. Colour indicates the study country and symbol indicates the migrant sub-population. Some studies report on more than one sub-population: in these cases only one is graphically depicted

Below, we give a review of individual diseases with the related literature. Figure 2 gives a graphical representation of the prevalence presented in studies on a general population of migrants including information on sub-population of migrant and country or region of origin in each study.

### Tuberculosis

#### Active tuberculosis

Approximately 25% of tuberculosis (TB) cases reported in EU in 2010 were found in foreign-born [18]. In Lebanon the TB incidence—decreasing from 1999 to 2006 but rising thereafter—reached a high of 20/100,000 in 2013 including a sharp rise in the proportion of multi-drug-resistant TB [19]. This rise was linked to the influx of Syrian refugees and Ethiopians in Lebanon. A study including a selected population of 44 Syrian refugees residing in a European country found a prevalence of 11% (*n* = 5) [8]. The prevalence in African asylum seekers in Malta during 2010 and 2011 was found to be 1%, in a migrant population primarily consisting of Pakistani and Afghan migrants presenting at the Greek-Turkish border during 2011 it was 8%, and in border-crossers in Europe 10% [7, 20, 21]. The latter study found TB to be the most prevalent infectious disease across border-crossers from all world regions with higher prevalence in people from South Asia and North and East Africa compared to those from South America and West Africa. In two studies from the USA, the prevalence of LTBI was high among refugees form Middle East (18%) and sub-Saharan Africa (43%) while active tuberculosis was rare [5, 22]. One study found 7 cases among 31,470 screened asylum seekers from Syria from 2011 to 2015 [23]. The authors conclude that this indicates that the WHO estimate of the country-specific TB incidence rate is a good approximation of the incidence in asylum seekers (for Syria this was 17/100,000 person years in 2014 [24]).

Chest X-ray may be the preferred method for active-TB screening of asylum seekers and refugees though a health interview has been proposed as a flexible and cost-cutting alternative [25, 26].

#### Latent tuberculosis

A CDC report found a latent tuberculosis infection (LTBI) prevalence of 14% in Iraqi refugees in the period October 2007 to September 2009 [27]. In a small Syrian study, the prevalence was 9% (*n* = 4) and in border-crossers in Europe 22% [7, 8]. Two studies from the USA found that the prevalence of LTBI was high among refugees from the Middle East (18%) and sub-Saharan Africa (43%) while active tuberculosis was rare [5, 22]. Predictors for LTBI among asylum seekers were as follows: origin from Africa or a former Soviet Union country, having travelled by land and coughing at presentation [28].

Most cases of TB in European countries are due to reactivation of LTBI acquired pre-entry to the host country with studies reporting a range of 5–72% of migrants testing positive for LTBI [29]. The risk of reactivation is highest in the years immediately after entry and decreases over time but remains increased compared to the autochthonous population probably due to a mixture of time of infection, poor living conditions in the host country, and considerable comorbidities and risk factors such as diabetes [22, 29–31].

The prevalence of multi-drug-resistant TB in migrants in Finland was found to be 2% with migrants from Somalia, Russia, and Estonia accounting for the vast majority of cases [31] and the TB mortality rate ratio in a group of all foreign-born compared with the autochthonous population in six European countries ranged between 0.56 (from East Asia) and 23 (from Latin America except Caribbean) with a rate ratio of 3 for foreign-born from North Africa [32]. The general decrease in TB incidence in European countries was not seen in the subgroup of foreign-born—thus the proportion of TB cases in foreign-born increased—and the socio-economic status of those infected differed between the autochthonous population and a group of foreign-born [18, 33].

The extent, the means, and the timing of screening of migrants are all subject of intense debate. One extreme is limiting TB screening to active disease post-entry. The other extreme is a very comprehensive screening effort pre-entry for LTBI including appropriate treatment. The latter has been implemented with success in some countries and may be cost-effective in populations from high-prevalence countries [17, 29, 34, 35]. A single blood test for LTBI, HIV, hepatitis B, and hepatitis C has been proposed as a means of raising the proportion of foreign-born that gets early diagnostics and treatment of these diseases in a general practitioner setting in London [36]. Compared with the existing system in 2014 (no formal screening programme for LTBI, hepatitis B, or hepatitis C), only LTBI was diagnosed more often but the results were seriously hampered by lack of participation. Chest X-ray may be the preferred method for active-TB screening of asylum seekers and refugees though a health interview has been proposed as a flexible and cost-cutting alternative [25, 26].

Some evidence point towards severe diagnostic delay and worse outcome of TB infection in the migrant population though there may be great diversity according to migrant status, time of residence in the host country, etc. [16]. Yet another study finds no difference in either diagnostic delay or outcome [37]. The tuberculin skin test (TST) is an affordable diagnostic for LTBI but means several visits to a health clinic. In a study of refugees attending a health clinic in the USA, more than half had a TST > 5 mm and most had a TST > 10 mm [38]. All patients were referred to a specialised unit but only half of the patients were followed up with a median of 50 days. With interferon-gamma release assays (IGRAs), it is possible to diagnose LTBI from one blood test. In a population of 541 refugees in the USA, one out of every four had a positive IGRA and almost all were asymptomatic [39]. In the same selected population mentioned above, only 61% of refugees from sub-Saharan Africa diagnosed with LTBI initiated treatment (79% of refugees from Middle East), another study found that only 1% of asylum seekers diagnosed with LTBI were treated and only after a long delay, and finally, a study of refugees in Australia found that particularly refugees from Eastern Mediterranean would refuse treatment—only 44% of all refugees diagnosed with LTBI completed treatment [22, 40, 41].

Important barriers to TB management in foreign-born populations are language and fear of deportation [37]. It must be stressed though, that even under very difficult conditions, such as in a refugee camp, it is possible to set up a strategy to ensure diagnostics and treatment. In a screening programme in a Jordanian refugee camp, 10% of the Syrian refugee population in Jordan were screened during the first 6 months of 2014 with X-ray and sputum examination if indicated and with a treatment adherence of 91% [42]. One study found seven cases among 31,470 screened asylum seekers from Syria from 2011 to 2015 [23]. The authors concluded that this indicate that the WHO estimate of the country-specific TB incidence rate is a good approximation of the incidence in asylum seekers (for Syria this was 17/100,000 person years in 2014 [24]).

### HIV

A small study conducted in late 2015 of 48 Syrian asylum seekers found no instances of infection with HIV, hepatitis B, or hepatitis C [43]. This is in line with the findings in a large study in the USA that found no instances of HIV infection in refugees from the Middle East and a prevalence of 3.3% in African refugees, a study that found a prevalence of 1% among all “border-crossers”, and CDC reporting of a prevalence of 0.7% in Iraqi refugees [5, 27, 44]. This was mirrored in a European setting by three studies: One study of migrants presenting at the Greek-Turkish border found only two cases (0.2% from Morocco and Iraq), a study found a prevalence of 1.5% in African refugees with a predominance of males, and finally a study found a prevalence of 4% in all asylum seekers in the UK [20, 45, 46]. Another study reported an overall prevalence of 7% for border-crossers in the EU, with the highest prevalence among East Africans (15%) and the lowest in South Asians (1%) [7]. In general, it has been found for all countries in the EU that the HIV incidence is higher among migrants than the autochthonous population. It is pointed out that restricted access to HIV prevention, testing, and treatment means that especially migrant sex workers are at risk [30]. As for tuberculosis, socio-economic status seems to be associated with the risk of HIV infection. Contrary to tuberculosis treatment, studies show that refugees have the same prevalence of sub-optimal adherence to HIV treatment as the autochthonous population [47].

### Hepatitis B

A study from Italy of 529 asylum seekers found 8.3% to be HBsAg positive and 45.6% to be anti-HBc positive [46]. This is remarkably higher than reported in a study from Germany on a population twice the size with predominantly migrants from Eastern Mediterranean, where numbers were 3.6 and 32.5%, respectively [48]. In a small study on 44 Syrian refugees, the prevalence for chronic HBV was 7% (3 cases) [8]. A review found the prevalence in asylum seekers in the UK to be between 6 and 12%, and other studies found it to be 12% for border-crossers into EU and 11% for all migrants in Spain, respectively [5, 45, 49]. In the USA, prevalence ranged between 11% (West Africa) and 2% (Eastern Europe) in both border-crossers and in a refugee population [5, 50]. There was some diversity in the reported association with region of origin: One study reported the highest prevalence among border-crosser from Southeast Asia and North Africa, and lowest in South Americans, while another study found a higher prevalence among sub-Saharan migrants [7, 49]. In a review from 2012, Asians had a high seroprevalence of HBV compared with other immigrants and there was an indication that refugee status may be an independent risk factor for HBV together with region of origin [51]. Compared with the autochthonous population in Italy, migrants with chronic HBV were found to be younger and predominantly females [52, 53]. Fewer immigrants had liver morbidities and fewer received antiviral treatment.

Although there is large variation of the prevalence, as well as the systems to survey and report cases of HBV between European countries, studies show that migrant populations from high endemic regions have increased prevalence compared to the autochthonous population [30]. In general, the migrants had a very low knowledge on hepatitis virus infection transmission routes [48] and one study found that only little more than one in ten eligible foreign-born were tested in the UK [54].

### Hepatitis C

In the study of 529 asylum seekers referred to above, 4.5% were anti-HCV positive, predominantly males and Asian refugees [46]. This is considerably higher than the 1.9% found in a German study and also higher than the one case that was found in a study of Syrian refugees in Europe but on par with another European study that found an overall prevalence of 5%, ranging from 1% in West and North Africans to 6% in East Africans [7, 8, 48]. A meta-analysis from 2015 found that—unlike for HBV—refugee status was not a risk factor for HCV [55]. Region of origin was, however, a strong risk factor particularly for individuals from sub-Saharan Africa, Asia, and Eastern Europe. The study found that migrants from one of these high endemic countries may benefit from targeted screening. In the Middle East, the prevalence in the general population was recently found to be very low [56] which indicates that HCV screening may not be a high priority in this large refugee population.

### Malaria

The prevalence of malaria among border-crossers was found to be 7% (highest among Southeast Asians and lowest in South Americans and North Africans) [7]. While malaria infection is very rare in the Middle East and North Africa, refugees from these regions often pass through countries where *Plasmodium vivax* transmission is possible, yet still rare, such as Greece and Turkey [57]. Conversely, refugees from sub-Saharan Africa and Asia could be infected before migration and there is a risk of (re-)introduction of the parasite in areas with competent vectors such as the *Anopheles* mosquito. It has been suggested that this is the likely explanation for the six cases of locally acquired *Plasmodium vivax* during the summer months in Greece in 2015 [57].

### Childhood diseases, vaccine preventable

Our search confirmed the lack of evidence on infectious diseases in refugee and asylum seeker children that has previously been pointed out [3, 30].

Children that are either refugees themselves or have parents who are refugees often lack routine vaccinations—either because of their parents’ unawareness of the vaccination programmes or because of unwillingness to participate [30]. Outbreaks of measles, rubella, and other childhood infections have been suggested to be associated with migration from low-coverage regions. In one study set at the Greek-Turkish border in 2011, 52.5% of migrant children needed vaccination against diphtheria, tetanus, and pertussis and 13.2% against measles, mumps, and rubella [20]. A study of refugee children under 5 years of age found the prevalence of rubella immunity to be 14% in African, 34% in Middle Eastern, 44% in Asian, and 71% in American refugee children [58]. The study found 50% to have measles immunity with no variation on world region.

### Other infectious diseases

#### Shigella

The ECDC have estimated the incidence of shigellosis to be 1.4/100,000 in 2014 in the EU/EAA, with the majority of infections (57%) to be travel-related. Several cases have been reported in refugees having the same migration route in common: through Turkey and Greece via the Balkans to Central Europe [59]. The ECDC concludes that it is not unexpected to see such cases given the hygienic conditions during the migration as well as in the reception facilities; furthermore, there is a high prevalence of shigella in many of the home countries and some of the countries the refugees travel through.

#### Cutaneous diphtheria

According to the ECDC, three European countries have reported a total of nine (seven toxigenic and two non-toxigenic) cases of cutaneous diphtheria in refugee populations in 2015 [60]. Since national health systems may have low sensitivity to cutaneous diphtheria among refugees due to the often limited access to health care, the number could be higher. Cutaneous diphtheria is a way for transmission of diphtheria. The high prevalence in many of the migrants’ country of origin combined with crowded and poor living conditions during and after migration are perfect conditions for the spread of diphtheria. Also, travellers that have not received vaccinations are at risk of infection.

#### Louse-born relapsing fever

There have been recent reports of 27 cases of louse-born relapsing fever among refugees taking the route through Libya to Italy and on to Central Europe [61]. The ECDC concludes that most cases developed in the home country or en route because of exposure to body lice. Yet, two cases were infected in Italy several years after arrival, probably due to shared living quarters with newly arrived asylum seekers. Again, the risk of infection is closely related to poor living conditions and there is very low risk of spread to the general population. Health care workers are also considered to be at low risk when taking the normal precautions such as wearing gloves during examination.

#### Leishmaniasis

There has been a steep rise in the number of cases of leishmaniasis among Syrian refugees in Lebanese refugee camps [62]. A total of 1033 cases were reported in 2013, of which 998 were Syrian refugees. Numbers from the first months of 2014 indicate no change from 2013. A recent study found that 32% (*n* = 14) of Syrian refugees in European countries had cutaneous leishmaniasis [8].

#### MRSA and ESBL/CPO

*Staphylococcus aureus* MRSA were isolated in rectal (2 of 3), pharyngeal (1 of 6), and nasal (3 of 16) swabs in Syrian refugees in Italy in 2015 [43]. In the same study, ESBL-producing gram-negative bacteria were found in rectal (6 of 27) and pharyngeal (1 of 5) swabs.

#### Sexually transmitted diseases

The prevalence of chlamydia was 3.3 and 1.4% in refugees from the Middle East and Eastern Europe, respectively, and 0.2% for gonorrhoea in refugees from sub-Saharan Africa and Southeast Asia [44]. In comparison, a study from the USA found a prevalence of 0% for both of these subgroups. The prevalence of syphilis was reported to be 2 and 1% for refugees from Africa and the Middle East, respectively [44, 46], and in a CDC report on adult Iraqi refugees, the prevalence was 2.6% [27]. In the same CDC report, *Giardia intestinalis* and *Entamoeba histolytica* were found with a prevalence of 3.1 and 1.2%, respectively.

## Conclusions

With this review, we have aimed to give a broad overview of many of the infectious diseases of concern in the refugee and asylum seeker populations in present time. We present the available literature on infectious diseases in *migrants*, with an effort to subdivide this very heterogeneous population, to be able to draw conclusions on important infectious diseases in the current refugee and asylum population.

The prevalence of tuberculosis rises during conflict—e.g. as seen in Iraq where the prevalence rose from 62/100,000 in 2000 to 74/100,000 in 2011 [42]—and is thus a concern in every asylum seeker and refugee population. We found latent tuberculosis to be the most prevalent infectious disease in the current asylum seeker and refugee population. Hepatitis B is another health concern for the current asylum seekers and refugees while both hepatitis C and HIV have low prevalence in this population. Chlamydia and syphilis were the most frequently reported sexually transmitted disease in this population. Malaria is very much related to the means and route of transportation as are a number of other infectious diseases that have been reported on a case basis but represent a risk of outbreaks due to reintroduction into areas where the disease has previously been eradicated, although only reported once.

Infectious diseases are among the significant health issues faced in the population of asylum seekers and refugees. The risk of transmission to the autochthonous population is very low, though outbreaks in the asylum seeker and refugee population should be considered due to poor living conditions and suboptimal vaccination, not least among children [4, 20, 63]. In late 2015, the ECDC published a set of recommendations including systems to ensure health assessment immediately after arrival to the host country, adequate living conditions, and free of charge access to diagnosis and treatment of any communicable disease [64]*.* A recent study found that little more than half of the EU countries have national or sub-national guidelines for screening of newly arrived migrants [15]. The most common screening program was targeted at tuberculosis screening and only a third of the EU countries screened for other infectious diseases such as hepatitis, HIV, or vaccine-preventable diseases. The most common place for screening was in asylum centres and only very few countries conducted screening at the pre-entry or entry stage of migration.

Few studies analysed data taking into account the reason of migration, the importance of which is illustrated by the possible association found between refugee status and HBV infection and the stronger evidence against such association to HCV [51, 55]. Most studies presented analysis accounting for world region of origin or did not consider ethnicity at all. While world region is preferable to the latter, this will still likely represent an extremely heterogeneous group in both risk epidemiology, reason for migration, and health knowledge.

The very broad scope of this review is a limitation, as it is not possible to provide an in-depth analysis of any one disease. Also, we have implemented a search strategy with MeSH terms exclusively. While this allows a high specificity in our search results, it may have excluded the most recently published articles. In our review we have included several studies on other migrant sub-populations than those of primary interest (refugees and asylum seekers) and even on the super-population “migrants”. We have done this to be able to present the best evidence on the subject at the moment and have taken great care to be specific on the sub-population in question.

The great diversity and often suboptimal reporting on the sub-population of migrants under study as well as the general lack of evidence in this area of research hampers inference on the health of asylum seekers and refugees and limits comparability across studies and countries. Published research on the health of a “migrant population” including all of foreign nationality should be clear on why such broad definition is warranted. While several studies include analysis of region of origin, and few studies do include migrant status as a factor in the analysis, the independent effect of fleeing and living as a refugee is still to be examined. The very different estimates of both HBV and HCV infection in the Italian and the German study is a good example of the difficulties of comparing or even reporting estimates for such heterogeneous groups [46, 48]. Even though the two countries are likely receiving migrants from the same areas (albeit set with 2 years apart), one study does not define migrants at all whereas the other defines migrants as asylum seekers; one chooses to group some countries, the other chooses another subdivision.

In a clinical setting, European countries should seek to accommodate this new and very heterogeneous subpopulation, e.g. by developing migrant health clinics that specialises to handle the health care needs of this diverse group as is seen for instance at the Odense University Hospital in Denmark [65]. This will help to strengthen the efforts already taking place in the receiving countries’ health care system and by a number of NGOs [3, 66]. The ECDC has developed a handbook for clinicians for health assessment of refugees and migrants in the EU/EEA [67]. Together with training of health care professionals, such initiatives are a step towards high quality and equal standards of health care in the reception of asylum seekers and refugees in the European countries.

As Clark and Mytton [45] put it “Without further healthcare services development and research, the prevalence of communicable diseases in asylum seekers and refugees will continue to remain the subject of speculation rather than fact. This will result in continuing policy development that is not evidence-based and insufficient treatment for this vulnerable sub-section of society.”

## Additional files

Additional file1: PRISMA-statement checklist with page number references. (PDF 109 kb)

## Abbreviations

Anti-HBc: Hepatitis B virus core antibody
Anti-HCV: Hepatitis C virus antibody
ESBL/CPO: Extended-spectrum beta-lactamase/carbapenemase-producing organism
HBsAg: Hepatitis B virus surface antigen
HBV: Hepatitis B virus
HCV: Hepatitis C virus
HIV: Human immunodeficiency virus
IGRA: Interferon gamma release assay
LTBI: Latent tuberculosis infections
MRSA: Methicillin-resistant *Staphylococcus aureus*
TB: Tuberculosis
TST: Tuberculin skin test
